# Transcriptome Analysis Unveils the Effects of Proline on Gene Expression in the Yeast *Komagataella phaffii*

**DOI:** 10.3390/microorganisms10010067

**Published:** 2021-12-29

**Authors:** Andrey Rumyantsev, Anton Sidorin, Artemii Volkov, Ousama Al Shanaa, Elena Sambuk, Marina Padkina

**Affiliations:** Laboratory of Biochemical Genetics, Department of Genetics and Biotechnology, Saint Petersburg State University (SPBU), 199034 Saint-Petersburg, Russia; antonsidorin@list.ru (A.S.); volkov.art.andr@gmail.com (A.V.); oalshanaa@mail.ru (O.A.S.); e.sambuk@spbu.ru (E.S.); m.padkina@spbu.ru (M.P.)

**Keywords:** *Pichia pastoris*, *Komagataella phaffii*, X-33, GS115, methanol metabolism, alcohol oxidase, *AOX1* promoter, proline

## Abstract

*Komagataella phaffii* yeast is one of the most important biocompounds producing microorganisms in modern biotechnology. Optimization of media recipes and cultivation strategies is key to successful synthesis of recombinant proteins. The complex effects of proline on gene expression in the yeast *K. phaffii* was analyzed on the transcriptome level in this work. Our analysis revealed drastic changes in gene expression when *K. phaffii* was grown in proline-containing media in comparison to ammonium sulphate-containing media. Around 18.9% of all protein-encoding genes were differentially expressed in the experimental conditions. Proline is catabolized by *K. phaffii* even in the presence of other nitrogen, carbon and energy sources. This results in the repression of genes involved in the utilization of other element sources, namely methanol. We also found that the repression of *AOX1* gene promoter with proline can be partially reversed by the deletion of the *KpPUT4.2* gene.

## 1. Introduction

One of the main aspects of modern biotechnology is the synthesis of peptides and recombinant proteins of various origins and for various applications. *Komagataella phaffii* (also known as *Pichia pastoris*) yeast is one of the most promising biotechnologically-relevant microorganisms, actively used as a microbial cell factory for large-scale recombinant protein production [1,2].

The successful use of *K. phaffii* in biotechnology, the pharmaceutical industry, and the food industry is largely due to the favorable characteristics of its metabolism. This yeast is methylotrophic, i.e., it is capable of using methanol as the sole carbon and energy source in the growth medium, thanks to a whole set of enzymes and their corresponding genes. The alcohol oxidase 1 (*AOX1*) gene encodes for an enzyme that catalyzes the oxidation of methanol into formaldehyde, representing the first reaction of the methanol utilization pathway in this microorganism [3]. *AOX1* gene expression is strictly regulated by the type of carbon source in the media [4]. For example, glycerol or glucose completely suppresses *AOX1* promoter activity. However, in the presence of methanol as the sole carbon source, *AOX1* gene expression is induced, with mRNA comprising up to 5% of the total mRNA in the cell. [3,4,5]. Due to its strict regulation and high activity, *AOX1* promoter is effectively used for the production of recombinant proteins in *K. phaffii* [1].

The biotechnological significance of *K. phaffii* and the active use of the *AOX1* promoter facilitated research on the molecular mechanisms underlying the regulation of *AOX1* gene and other genes involved in methanol metabolism (Methanol UTilization, *MUT* genes) [6]. A number of transcription factors that directly bind to *MUT* promoters and regulate their activity has been found. Mxr1 [7], Mit1 [8] and Trm1 [9] proteins upregulate *AOX1* gene transcription, while Mig1, Mig2 [10], Rop1 [11] and Nrg1 [12] downregulate *AOX1* gene transcription. In fact, these transcription factors are also targets of other components of regulatory systems. For instance, 14-3-3 type protein binds to Mxr1 and inhibits its activity [13]. The role of various kinases in the regulation of the *AOX1* gene has also been demonstrated [14].

Proteins that are indirectly involved in the regulation of *MUT* genes have also been found, like the membrane transporter proteins Gt1 and Hxt1. Gt1 protein is a glycerol transmembrane transporter. Therefore, Gt1 gene deletion results in *AOX1* derepression in glycerol-containing media [15]. Moreover, *K. phaffii* has two hexose transporter homologs, namely Hxt1 and Hxt2. *PpHXT1* gene deletion leads to the induction of *AOX1* gene expression in response to glucose or fructose, giving rise to reliable methanol-free expression systems [16].

The current published literature mainly focuses on the regulation of *MUT* genes by carbon sources such as glycerol, methanol, and glucose. At the same time, the expression of *MUT* genes has also been found to be influenced by other components of the growth medium. We have previously shown that when the culture medium contains certain amino acids as the only nitrogen sources, the activity of *AOX1* gene promoter and other *MUT* genes is suppressed [17,18]. This type of regulation of *MUT* genes may be due to the fact that *K. phaffii* yeast is capable of using a number of amino acids as the only carbon and energy source [19,20]. It has been shown that Mxr1 protein, the main inducer of *MUT* genes, also regulates the synthesis of key enzymes involved in the utilization of amino acids [19].

The highest level of *MUT* repression by amino acids was observed during the growth of *K. phaffii* in proline-containing media [17]. Proline has multiple functions in living cells. First, it is one of the building blocks of proteins. Proline is also a signaling molecule in yeasts. For example, it has been reported that intracellular proline is able to regulate the replicative lifespan in the baker’s yeast *Saccharomyces cerevisiae* [21]. Another function of this amino acid is helping the yeast cells to cope with various stressors, and its accumulation increases the resistance of yeast cells to freezing, drying, oxidation, and the toxic action of ethanol [22]. At the same time, proline stabilizes membrane proteins, participates as a chaperone in protein folding, and neutralizes reactive oxygen species. Proline can be used by *K. phaffii* as a sole source of nitrogen and carbon [20]. In general, proline and arginine are the main natural substrates for yeast living on grapes [23].

Proline catabolism and its regulation have been studied in detail in the yeast *S. cerevisiae*. The intracellular level of proline in this microorganism is largely determined by the activity of permeases—Gap1, Agp1, Gnp1 and Put4 [24]. Gap1 is a transporter for all naturally occurring amino acids [25]. Permeases Agp1 and Gnp1 also have broad substrate specificity [26,27]. However, Put4 protein is highly specific and only transports proline [28].

Once in the yeast cell, proline is transported into the mitochondria, where it is converted to delta-1-pyrroline-5-carboxylate (P5C) by proline oxidase encoded in *S. cerevisiae* by the *PUT1* gene. P5C is then converted to glutamate by delta-1-pyrroline-5-carboxylate dehydrogenase, encoded by the *PUT2* gene [29,30]. Furthermore, glutamate can be converted to alpha-ketoglutarate, which is a part of the tricarboxylic acid (TCA) cycle. An alternative pathway for the degradation of glutamate begins with its conversion to gamma-aminobutyric acid, and then into succinate, which is also a part of the TCA [31,32].

The aim of this research is to study the complex effects of proline on gene expression in the yeast *K. phaffii*.

## 2. Materials and Methods

### 2.1. Yeast and Bacterial Strains

*K. phaffii* strains used in this study are listed in Table 1.

The bacterial strain *Escherichia coli* DH5α (*F′phi80dlacZ delta (lacZYA_argF) U169 deoRrecA1 endA1 hsdR17 (rK–mK+) phoA supE44lambda_thi_1 gyrA96 relA1/F′ proAB+ lacIqdeltaM15 Tn10* (*tetr*)) (Thermo Fisher Scientific, Waltham, MA, USA) was used for the construction of plasmids.

### 2.2. Media and Cultivation Conditions

Standard LB medium was used for *E. coli* manipulations. LB contained (here and further *w/v*) 1% tryptone, 0.5% yeast extract, 1% NaCl, and 2.4% agar for plates.

YPD media was used for routine manipulations with *K. phaffii* strains. YPD contained 2% glucose, 2% peptone, 1% yeast extract, and 2.4% agar. YPDS media was used for zeocin selection. YPDS contained 2% glucose, 2% peptone, 1% yeast extract, 18.2% sorbitol (1 M), 2.4% agar, and 200 mg/L zeocin.

Modifications of minimal media were used for the selection of transformed *K. phaffii* cells, growth studies, and qualitative enzymatic assays. Each type of media contained: 1.34% yeast nitrogen base without amino acids and without ammonium sulphate, and 2.4% agar with either 1% glucose, 1% glycerol or 1% methanol added separately as carbon sources. Similarly, 0.46% ammonium sulphate, 0.46% proline or their mix were added separately as nitrogen sources.

Modifications of standard buffered complex media were used for cultivating *K. phaffii*, transcriptome analysis, and quantitative enzymatic assays. Each type of media contained 100 mM potassium phosphate, pH 6.0, 4 × 10^−5^% biotin and 1.34% yeast nitrogen base without amino acids and without ammonium sulphate. BMGN medium contained 1% glycerol and 0.46% ammonium sulphate. BMGP medium contained 1% glycerol and 0.46% proline. BMMN medium contained 0.5% methanol and 0.46% ammonium sulphate. BMMP medium contained 0.5% methanol and 0.46% proline. Yeast cells were grown at 30 °C and bacterial cells at 37 °C.

### 2.3. Oligonucleotides

Primer sequences used in this study are listed in Table S1 in Supplementary Materials.

### 2.4. Molecular Methods

Yeast and bacterial cell transformations were carried out using electroporation in accordance with [33,34].

Plasmid extraction was performed using the Plasmid Miniprep kit (cat.# BC021S, Evrogen, Moscow, Russia). Yeast genomic DNA extraction was performed using LumiPure from AnySample kit (cat.# 21573, Lumiprobe, Moscow, Russia). Purification of DNA from agarose gels was carried out using the Cleanup Standard kit (cat.# BC022, Evrogen, Moscow, Russia).

DNA digestion by restriction endonucleases, dephosphorylation of vectors and DNA ligation were performed using the buffers and conditions recommended by the manufacturer of the enzymes (restriction enzymes: SacI cat.# ER1131, AvrII cat.# ER1561, BglII cat.# ER0081, BamHI cat.# ER0051, and alkaline phosphatase FastAP cat.# EF0651, Thermo Fisher Scientific, Waltham, MA, USA). Routine PCR was performed using the Encyclo Polymerase kit (cat.# PK001, Evrogen, Moscow, Russia). In case the amplified DNA fragments were to be used in further cloning, the Thersus Polymerase kit (cat.# PK121, Evrogen, Moscow, Russia) was used. DNA electrophoresis was performed according to [35].

### 2.5. Enzymatic Assays

Qualitative determination of acid phosphatase (ACP) activity was carried out according to the standard method developed earlier [36]. First, the filter paper was dipped in a solution of substrate α-naphthyl phosphate and Fast Blue B at a concentration of 2 mg per mL of 0.1 M citrate buffer, pH 4.6. Second, the filter paper was applied to the surface of the agar medium with grown yeast colonies. After 10–15 min, the staining intensity of the yeast colonies was assessed.

Quantitative determination of ACP was carried out according to [37]. 100 μL of yeast cell suspension was taken and added into 800 μL of 0.1 M Na-citrate buffer, pH 4.6. Next, 100 μL of 0.15 M p-nitrophenyl phosphate substrate was added. The reaction mixture was stirred and incubated for 20 min at 30 °C in a water bath. The reaction was stopped by adding 500 μL of 1 M NaOH. The light absorption of the solutions was measured at a wavelength of 410 nm. The specific activity of ACP was determined as the ratio of the absorption of light by the reaction mixture at a wavelength of 410 nm to the optical density of the initial cell suspension at a wavelength of 550 nm.

We modified 1-(4,5-dimethylthiazol-2-yl)-3,5-diphenyltetrazolium bromide (MTT) assay according to [38]. We added 50 μL of 20 mM MES to 50 μL of yeast cultures. We added 10 μL of 4 mg/mL MTT solution and the samples were mixed thoroughly. The reaction mixtures were incubated for 30 min at 30 °C. After incubation, the cells were collected by centrifugation and supernatant was aspirated. We added 100 μL of DMSO to yeast pellets and the samples were mixed thoroughly. The determination of the MTT reduction was performed using a microplate reader at 570 nm. The specific activity was determined as the ratio of the absorption of light by the reaction mixture at a wavelength of 570 nm to the optical density of the initial cell suspension at a wavelength of 550 nm.

### 2.6. Gene Nomenclature

For annotated *K. phaffii* genes (*AOX1*, *AOX2* etc.) names were acquired from published studies, especially [39]. Other *K. phaffii* genes that are orthologs of the *S. cerevisiae* genes were found using BLAST analysis [40] (Table S5 in Supplementary Materials). “*Kp*” index was added to the name of these genes in order to distinguish them from *S. cerevisiae* ones.

### 2.7. The Construction of K. phaffii P1AP-GS115 Strain

*S. cerevisiae* Put1 amino acid sequence (NP_013243.1) was used as the query for BLAST analysis [40]. Proline oxidase (XP_002489614.1) was found among *K. phaffii* GS115 (taxid:644223) proteins. The corresponding *K. phaffii* gene (PAS_chr1-3_0269), which is orthologous to the *S. cerevisiae PUT1* gene, is named *KpPUT1*.

The first DNA fragment corresponding to *KpPUT1* gene promoter was PCR amplified with PPUT1-F-SacI and PPUT1-R primers and genomic DNA from *K. phaffii* GS115 strain as the DNA template. The second fragment corresponding to *S. cerevisiae PHO5* acid phosphatase gene was PCR amplified with PHO5-F and PHO5-R-AvrII primers and pPIC9-PHO5 plasmid [17,18] as a template. The fragments were purified by agarose gel electrophoresis. Their mix was used as a template for overlapping PCR with PPUT1-F-SacI and PHO5-R-AvrII primers. The resulting fragment was cloned into pPIC9 plasmid using SacI and AvrII restriction sites. pPIC9-PPUT1-PHO5 plasmid was linerialized using StuI restriction enzyme and transformed into *K. phaffii* GS115 *his4* strain. The resulting P1AP-GS115 strain carries *PHO5* reporter gene under the control of *KpPUT1* gene promoter. The strain was checked using PCR (Figure S1a in Supplementary Materials).

### 2.8. The Introduction of Gene Deletions in K. phaffii

*S. cerevisiae* Gap1 (NP_012965.3) and Put4 (NP_014993.1) amino acid sequences were used as the query for BLAST analysis [40]. Homologous sequences were found among *K. phaffii* GS115 (taxid:644223) proteins. Gene deletions were introduced in corresponding *K. phaffii* genes: *KpGAP1.1* (*PAS_chr1-4_0479*), *KpGAP1.2* (*PAS_chr1-1_0203*), *KpGAP1.3* (*PAS_chr1-1_0030*), *KpPUT4.1* (*PAS_chr2-1_0129*), and *KpPUT4.2* (*PAS_chr2-1_0659*).

Separate DNA fragments corresponding to 5′ and 3′ homology arms (HA) of the targeted genes were amplified using PCR with specific primer pairs (Table S1) and the genomic DNA from *K. phaffii* GS115 strain as a template. Fragments were purified by agarose gel electrophoresis. For each gene a mix of two corresponding fragments (5′-HA and 3′-HA) was used as DNA template for overlapping PCR with flanking primers (-5F and -3R). The resulting 5′-3′-HA fragments contained BamHI restriction site that was introduced via the primers between 5′-HA and 3′-HA parts. Fragments were cloned into pAL2-T plasmid (cat.# TA002, Evrogen, Moscow, Russia) using the Quick-TA kit (cat.# TAK02, Evrogen, Moscow, Russia). The pPICZαA (cat.# V195-20, Thermo Fisher Scientific, Waltham, MA, USA) plasmid was cut using BglII and BamHI restriction enzymes. A fragment containing zeocin resistance gene (ZeoR) was purified by agarose gel electrophoresis. It was cloned inside pAL2-T-5′-3′-HA plasmids using BamHI restriction site.

The resulting plasmids (pKpGAP1.1-5′-ZeoR-3′, pKpGAP1.2-5′-ZeoR-3′, pKpGAP1.3-5′-ZeoR-3′, pKpPUT4.1-5′-ZeoR-3′ and pKpPUT4.2-5′-ZeoR-3′) were used as templates for PCR with flanking primers (-5F and -3R). The PCR fragments were used for the transformation of *K. phaffii* tr2-1-GS115 strain, which contains a *PAOX1-PHO5* reporter construction [17,18]. Thus, *K. phaffii* Δgap1.1-GS115, Δgap1.2-GS115, Δgap1.3-GS115, Δput4.1-GS115 and Δput4.2-GS115 strains were obtained. These strains carry deletions in *K. phaffii KpGAP1.1*, *KpGAP1.2*, *KpGAP1.3*, *KpPUT4.1* or *KpPUT4.2* genes, and the *PHO5* reporter gene under the control of the *AOX1* gene promoter. The strains were checked using PCR (Figure S1b in Supplementary Materials).

### 2.9. RNA-Sequencing

Total RNA was isolated from cells using the RNeasy Mini Kit (cat.# 74104, Quiagen, Germantown, MD, USA). After isolation, DNase (cat.# EN0525, Thermo Fisher Scientific, Waltham, MA, USA) treatment was performed to remove DNA molecules from the sample. Agarose gel electrophoresis was performed to assess the quality of the isolated RNA molecules.

Further preparation and sequencing was done using the TruSeq mRNA Stranded reagent kit (cat.# 20020594, Illumina, San Diego, CA, USA). The quality of total RNA was verified by horizontal gel electrophoresis, the total RNA samples were enriched for the poly(A+) fraction, and then cDNA was synthesized from the random primers. The resulting cDNA was used to prepare libraries compatible with Illumina sequencing technology.

The quality of the libraries was checked using the Fragment Analyzer 5200 system (Agilent Technologies, Santa Clara, CA, USA) and Small Fragment Kit, 500 (cat.# DNF-476-0500, Agilent Technologies, Santa Clara, CA, USA). Quantitative analysis was performed using the qPCR method. After quality control and estimation of the DNA amount, the pool of libraries was sequenced on Illumina MiSeq (read length—300 bp on one side of the fragments).

FASTQ files were obtained using bcl2fastq v2.20 Conversion Software (Illumina, San Diego, CA, USA). The format for recording the quality data line is Phred 33. General information on the number of reads is presented in Table S2.

### 2.10. Bioinformatic Analysis

Quality of reads was assessed using the fastqc program [41]. The Trimmomatic software [42] removed the adapter sequences and filtered the reads by quality. The sequence and annotation of the reference genome of *K. phaffii* (ASM2700v1) were taken from the NCBI database. The reads were aligned with the reference genome using the hisat-2 program [43] with standard parameters.

The number of aligned reads per gene was counted using the featureCounts program [44]. Then we used the R programming language (version 3.6.3) to statistically evaluate the difference in the resulting counts (differential gene expression analysis). This analysis was performed using the DESeq2 package (version 1.24.0) [45]. For further analysis we took differentially expressed genes (DEG) with values of the logarithm of the change in the amount of expression (log2FoldChange) greater than 0.5 or less than –0.5. The value of the adjusted *p*-value (*p* adj value) for analyzed DEG was less than 0.05. The results of the DESeq2 are presented in Tables S3 and S4.

## 3. Results

### 3.1. The Analysis of KpPUT1 Gene Expression in K. phaffii

In *S. cerevisiae* yeast, the first reactions of proline utilization occur in the mitochondria. These reactions are catalyzed by proline oxidase and delta-1-pyrroline-5-carboxylate dehydrogenase, encoded by *PUT1* and *PUT2* genes, respectively [29]. In this work, we studied the expression of *KpPUT1* gene (PAS_chr1-3_0269) in *K. phaffii* yeast, which is the ortholog of the *S. cerevisiae PUT1* gene.

**Figure 1:**
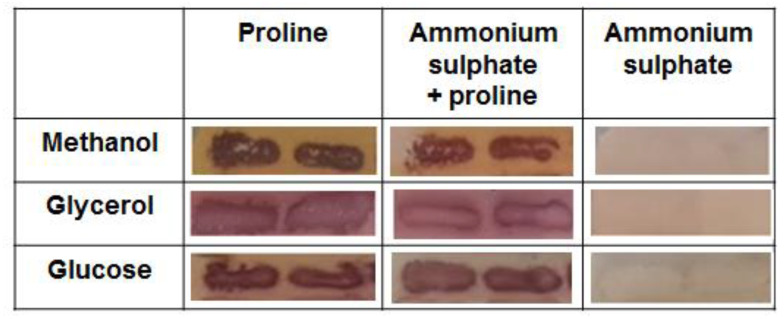
Photographs of *K. phaffii* P1AP-GS115 strain colonies grown on media with different carbon and nitrogen sources illustrate the results of the qualitative analysis of ACP reporter system activity.

*K. phaffii* P1AP-GS115 strain has been constructed in our lab. This strain has the *PHO5* acid phosphatase (ACP) gene from *S. cerevisiae* under the control of the *KpPUT1* gene promoter, creating a reliable and robust reporter system. This strain was grown on plates with minimal media. In fact, phosphate in the media inhibits the activity of the native *K. phaffii* ACP genes (e.g., *PHO1*). Thus, the observed ACP activity on the cell surface in this strain is the result of the *KpPUT1* gene promoter activity in the reporter system. Methanol, glycerol and glucose were used as the main carbon sources. Ammonium sulphate, proline, and their mixes were used as the main nitrogen sources. ACP reporter activity on the surface of the colonies was determined qualitatively after 48 h of growth (Figure 1).

Thus, we demonstrated the activation of the *K. phaffii KpPUT1* gene promoter in proline-containing media, with minor differences in the results due to the different types of carbon sources used in the growth medium, or the presence/absence of ammonium sulfate. Along with the previously shown ability of *K. phaffii* to grow on media with proline as the only source of carbon and nitrogen [20], our findings suggest that active catabolism of proline in *K. phaffii* cells occurs even in the presence of other carbon and nitrogen sources. Therefore, we further investigated how proline utilization affected gene expression in this yeast.

### 3.2. The Analysis of Gene Expression of K. phaffii Yeast on the Transcriptome Level

Our previous studies mainly used the *K. phaffii* GS115 *his4* strain and its derivatives [17,18,20]. The GS115 strain requires histidine for growth, while the presence of different amino acids may result in the repression of *MUT* genes [17,19,20]. Therefore, wild-type X-33 strain was chosen for transcriptome analysis experiments in this study. This also allowed comparing the effects of proline on these two *K. phaffii* strains that are widely used in biotechnology.

The experiment was based on a two-stage cultivation scheme often used for recombinant protein production in K. phaffii. In the first stage, K. phaffi X-33 strain was grown in 100 mL of glycerol-containing media with ammonium sulfate (BMGN) or proline (BMGP). After 40 h of growth (in the incubator shaker at 100 rpm) the cells were collected and transferred into 100 mL of methanol-containing media with ammonium sulfate (BMMN) or proline (BMMP). After 15 h of methanol induction, total RNA was extracted from the cells and used for RNA sequencing.

RNA sequencing data analysis revealed the expression of 4920 protein encoding genes, of which 929 (18.9%) were differentially expressed genes (DEG) (Table S3); 554 genes of the total DEG demonstrated increased expression in cells cultured in proline-containing media, in comparison with cells cultured in ammonium sulfate-containing media. Accordingly, 375 genes were identified, the expression of which decreased in proline-containing media. In this paper we mainly focused on methanol metabolism genes (*MUT* genes) and the genes that are presumably involved in proline metabolism in *K. phaffii* (Table S4).

**Figure 2:**
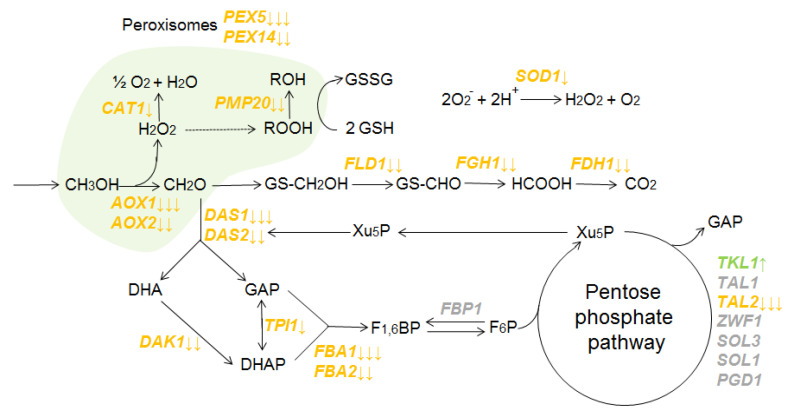
Scheme of the canonical methanol utilization pathway in *K. phaffii* cells (modified from [39]). Genes are placed near the reactions catalyzed by the correspondent proteins. Peroxisomal genes are also shown. Genes that are repressed in media with proline are marked in orange. Genes that are activated in media with proline are marked in green. Genes that do not change their expression are marked in grey. The number of arrows represents values of log2FoldChange parameter. One arrow: 0.5 < log2FoldChange < 1; two arrows: 1 < log2FoldChange < 2; three arrows: 2 < log2FoldChange. Abbreviations of metabolites: DHA, dihydroxyacetone; DHAP, dihydroxyacetone phosphate; FRU_1,6_BP, fructose-1,6-bisphosphate; F_6_P, fructose-6-phosphate; GAP, glyceraldehyde-3-phosphate; GSH, glutathione; GSSG, oxidized glutathione self-dimer; Xu5P, xylulose 5-phosphate.

A decrease in the activity of *MUT* genes was observed when *K. phaffii* was cultured in proline-containing media in comparison to ammonium sulfate-containing media (Figure 2). Regulation with proline affects all stages of methanol metabolism. The expression of alcohol oxidases genes (*AOX1*, *AOX2*) is decreased along with genes involved in the dissimilative branch (*FLD1*, *FGH1*, *FDH1*) and the assimilative branch of the methanol metabolic pathway (*DAS1*, *DAS2*, *DAK1*, *TPI1*, *FBA2*). mRNA levels also decreased for genes involved in the cellular defense mechanisms against ROS (reactive oxygen species), which are normally produced during methanol oxidation (*CAT1*, *SOD1*, *PMP20*). Thus, when *K. phaffii* is cultured in proline-containing media, the expression of known *MUT* genes, which are involved in all stages of methanol metabolism, is suppressed at the transcriptional level.

The expression of PPP (pentose phosphate pathway) genes is inextricably linked to methanol metabolism, and it changed in different ways in our experiments. Transcription of genes involved in the oxidative phase of PPP did not change significantly (*ZWF1*, *SOL3*, *SOL1*, *PGD1*). However, the genes involved in the non-oxidative phase of PPP responded differently. When cultured in proline-containing media, the expression rate of the transketolase gene (*TLK1*) increased and the expression rate of the transaldolase gene (*TAL2*) decreased.

The expression of genes involved in the biogenesis and functioning of peroxisomes (*PEX* genes) was also analyzed. We found that when *K. phaffii* was cultured in proline-containing media, the expression level of the main *PEX* genes (e.g., *PEX5*, *PEX14*) decreased.

Interestingly we also found that expression of *MIG2* gene (PAS_chr1-4_0526), which is involved in the repression of *AOX1* and other *MUT* genes [10] is significantly activated when *K. phaffii* was cultured in proline-containing media. *K. phaffii* gene *PAS_chr2-1_0639*, which is annotated in the NCBI database as an upstream serine/threonine kinase for the SNF1 complex, also demonstrates increased gene expression in the presence of proline. The amino acid sequence of the corresponding protein (XP_002491563.1) in *K. phaffii* is homologous to Sac1, Tos3 and Elm1 in *S. cerevisiae*.

Next, we analyzed the expression of genes that were presumably involved in proline metabolism in *K. phaffii*. First, genes that may be involved in proline transport into the cell were investigated. Three *K. phaffi* genes were found to be presumably orthologs of the *GAP1* gene, which, in turn, encode for the general amino acid permease in *S. cerevisiae*. They were named *KpGAP1.1* (PAS_chr1-4_0479), *KpGAP1.2* (PAS_chr1-1_0203) and *KpGAP1.3* (PAS_chr1-1_0030). The expression of *KpGAP1.1* and *KpGAP1.2* genes was higher in proline-containing media in comparison to ammonium sulfate-containing media. Expression of the *KpGAP1.3* gene, on the contrary, decreased in response to the presence of proline.

Two orthologous genes to the specific proline permease gene *PUT4* in *S. cerevisiae* were also found. They were named *KpPUT4.1* (PAS_chr2-1_0129) and *KpPUT4.2* (PAS_chr2-1_0659). Expression of the *KpPUT4.1* gene occurred at a relatively low level and did not differ significantly during cultivation in ammonium sulfate-containing media in comparison to proline-containing media. At the same time, the *KpPUT4.2* gene showed significant changes in expression levels. It was actively transcribed during the cultivation of *K. phaffii* in proline-containing media and was practically inactive in ammonium sulfate-containing media.

In *S. cerevisiae* yeast, the degradation of proline by proline oxidase Put1 and delta-1-pyrroline-5-carboxylate dehydrogenase Put2 leads to the formation of glutamate. The RNA sequencing data confirmed the activation of the *KpPUT1* gene in proline-containing media. The *KpPUT2* gene, which is presumably an ortholog of the *S. cerevisiae PUT2* (PAS_chr2-2_0496) gene, was also actively transcribed in the presence of proline, and was suppressed when *K. phaffii* was cultured in the presence of ammonium sulfate.

In *S. cerevisiae* the glutamate formed during the oxidation of proline can be converted into α-ketoglutarate by the NAD(+)-dependent glutamate dehydrogenase, encoded by the *GDH2* gene [31]. An alternative degradation pathway leads to the conversion of glutamate to gamma-aminobutyric acid (GABA) by glutamate decarboxylase, encoded by *GAD1* gene. GABA is then converted to succinate. This process is controlled by GABA transaminase and succinate semialdehyde dehydrogenase, encoded by *UGA1* and *UGA2* genes, respectively [32]. The results of RNA sequencing demonstrate an increase in the expression of *KpGDH2* (PAS_chr2-1_0311), *KpGAD1* (PAS_chr3_0965), *KpUGA1.2* (PAS_chr2-1_0107) and *KpUGA2* (PAS_chr1-3_0024) genes, when *K. phaffii* is cultured in media with proline.

**Figure 3:**
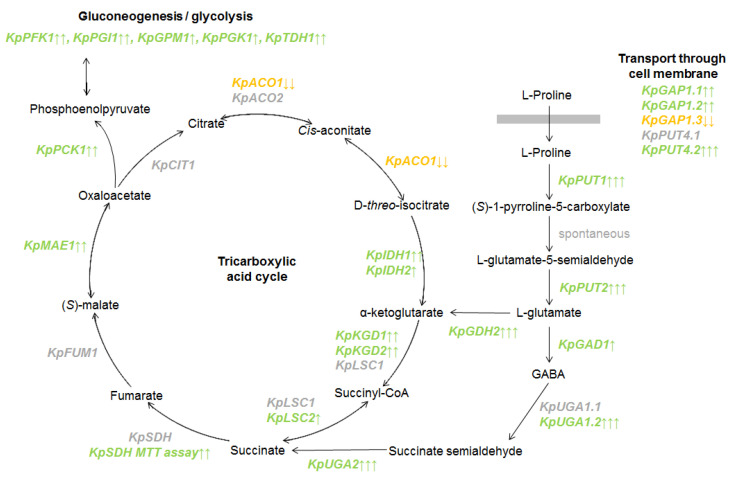
Scheme representing proline oxidation, gamma-aminobutyric acid (GABA) shunt and tricarboxylic acid (TCA) (based on data known for *S. cerevisiae*). Genes are placed near the reactions catalyzed by the correspondent proteins. Genes that are repressed in media with proline are marked in orange. Genes that are activated in media with proline are marked in green. Genes that do not change their expression are marked in grey. The number of arrows represent values of log2FoldChange parameter. One arrow: 0.5 < log2FoldChange < 1; two arrows: 1 < log2FoldChange < 2; three arrows: 2 < log2FoldChange.

α-Ketoglutarate and succinate are both components of the TCA cycle. Therefore, we analyzed the expression of genes that are presumably orthologs of genes for TCA enzymes in *S. cerevisiae*. During the cultivation of the yeast *K. phaffii* in proline-containing media, significant changes in the expression of these genes were observed (Figure 3).

Expression of the *KpCIT1* (PAS_chr1-1_0475) gene encoding citrate synthase did not change significantly. The aconitase *KpACO1* (PAS_chr1-3_0104) gene is repressed in proline-containing media. A second aconitase gene *KpACO2* (PAS_chr3_0659) did not change its expression. It was previously shown that in respiring filamentous fungi *ACO2* orthologs are enzymatically inactive [46].

Expression of the *KpIDH1* (PAS_chr4_0580) and *KpIDH2* (PAS_chr2-1_0120) genes presumably encoding NAD(+)-dependent isocitrate dehydrogenase was higher in proline-containing media. Two of the genes, that presumably encode α-ketoglutarate dehydrogenase complex, *KpKGD1* (PAS_chr2-1_0089) and *KpKGD2* (PAS_chr1-3_0094), also demonstrate an increase in the expression in proline-containing media, while expression of *KpLPD1* (PAS_chr2-2_0048) encoding the lipoamide dehydrogenase component of the complex did not change significantly.

The *KpLSC1* (PAS_chr3_0831) gene encoding α-subunit of succinyl-CoA ligase did not change its expression, while the *KpLSC2* (PAS_chr2-2_0407) gene encoding β-subunit was more active in proline-containing media. Expression of the mitochondrial malic enzyme gene *KpMAE1* (PAS_chr3_0181) was also higher in proline-containing media.

Fumarate hydratase gene *KpFUM1* (PAS_chr3_0647) along with succinate dehydrogenase genes *KpSDH* (PAS_chr2-2_0283 and PAS_chr3_0424) did not show any significant change in their expression. Since gene expression can be regulated on different levels, an enzymatic assay was used to measure the activity of succinate dehydrogenase.

Transcriptome analysis also showed that in *K. phaffii* cells a significant number of genes involved in glycolysis and gluconeogenesis demonstrated an increase in the expression in proline-containing media. First, the *KpPCK1* (PAS_FragB_0061) gene which encodes phosphoenolpyruvate carboxykinase—a key enzyme in gluconeogenesis, catalyzes early reaction in carbohydrate biosynthesis. Other genes, activated by proline, presumably encode for phosphofructokinase (*KpPFK1*, PAS_chr2-1_0402), phosphoglucose isomerase (*KpPGI1*, PAS_chr3_0456), phosphoglycerate mutase (*KpGPM1*, PAS_chr3_0826), 3-phosphoglycerate kinase (*KpPGK1*, PAS_chr1-4_0292) and glyceraldehyde 3-phosphate dehydrogenase (*KpTDH1*, PAS_chr2-1_0437).

### 3.3. The Analysis of the Mitochondrial Activity in K. phaffii Cells

The MTT assay is commonly used to evaluate mitochondrial activity and functionality in cultured cell lines [47,48]. Cleavage of MTT in active mitochondria results in formazan product accumulation, and is performed in yeast mostly by the succinate dehydrogenase [38,49].

**Figure 4:**
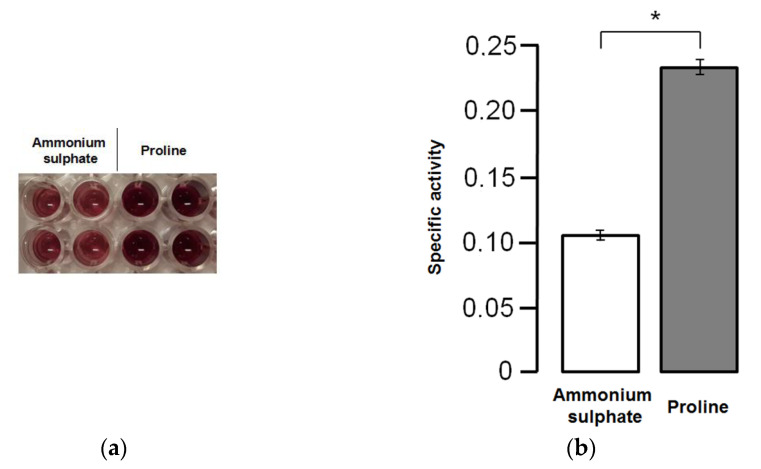
The effect of proline on the mitochondrial activity in the cells of *K. phaffii* X-33 strain: (**a**) image of the MTT assay results; (**b**) bar chart representation of mitochondrial activity in the cultures of X-33 strain after growth and methanol induction in media with ammonium sulphate or proline. The mean with SEM from four separate experiments with two technical replications is presented (* *p* < 0.01; Mann–Whitney U test).

To analyze the mitochondrial activity in *K. phaffii* X-33 strain, cells were grown in 100 mL glycerol-containing media with ammonium sulfate (BMGN) or proline (BMGP). After 40 h of cultivation, the cells were collected and placed in 100 mL methanol-containing media with ammonium sulfate (BMMN) or proline (BMMP). After 40 h of methanol induction, the MTT assay was performed (Figure 4).

We showed that mitochondrial succinate dehydrogenase activity increased when *K. phaffii* was cultured in the medium containing methanol and proline in comparison to the medium containing methanol and ammonium sulphate. Although succinate dehydrogenase genes (*KpSDH*) did not show any significant change in mRNA levels, the results of the enzymatic assay demonstrate that their expression is induced by proline at a post-transcriptional level.

### 3.4. The Analysis of the Phenotypes of K. phaffii Strains Carrying the Deletions in Permease Genes

It was shown that deletions of genes encoding for membrane transporter proteins of glycerol (*GT1*) and hexoses (*PpHXT1*) led to the removal of the repression of the *AOX1* promoter by glycerol and glucose [15,16]. We hypothesized that impairment of proline transport into cells may affect the regulation of the *AOX1* gene promoter by this amino acid.

*K. phaffii* strains that are carrying deletions in *KpGAP1.1*, *KpGAP1.2*, *KpGAP1.3*, *KpPUT4.1*, and *KpPUT4.2* were developed. These strains also carry the *S. cerevisiae PHO5* reporter gene under the control of the *AOX1* gene promoter. These strains (Δgap1.1-GS115, Δgap1.2-GS115, Δgap1.3-GS115, Δput4.1-GS115, Δput4.2-GS115) along with the control strain (tr2-1-GS115) were grown on minimal media with glycerol or methanol. Ammonium sulfate or proline was used as the only nitrogen source. Under the studied conditions, the growth of strains containing deletions did not significantly differ from the growth of the control strain (Figure S2a in Supplementary Materials).

In *S. cerevisiae*, citrulline transport is carried out by the general amino acid permease Gap1. Thus, it is impossible for *S. cerevisiae* strains with a deletion of *GAP1* gene to grow on a medium with this amino acid as a sole nitrogen source [50]. Interestingly, the deletions in either *KpGAP1.1*, *KpGAP1.2* or *KpGAP1.3* genes did not impair the growth of *K. phaffii* strains on media with citrulline as the only nitrogen source (Figure S2b in Supplementary Materials).

### 3.5. The Analysis of the Effects of the Deletions in Permease Genes on the AOX1 Promoter Activity

**Figure 5:**
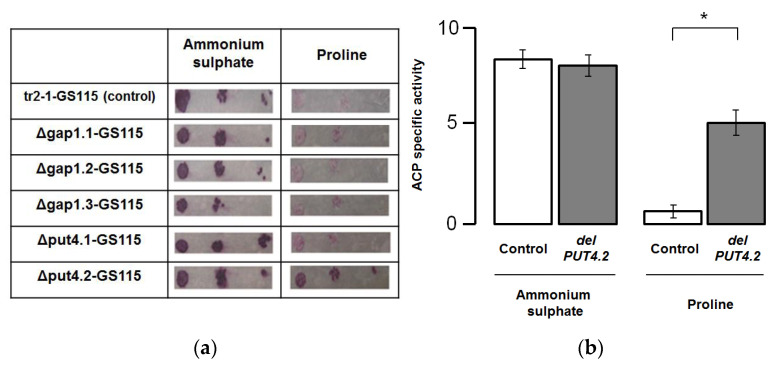
Effect of deletions in the *K. phaffii* permease genes on the activity of the *PHO5* ACP reporter gene under the control of the *AOX1* gene promoter: (**a**) results of qualitative analysis of ACP activity on the surface of the colonies of Δgap1.1-GS115, Δgap1.2-GS115, Δgap1.3-GS115, Δput4.1-GS115, Δput4.2-GS115 and control tr2-1-GS115 strains grown on plates with minimal media containing methanol in addition to either ammonium sulphate or proline; (**b**) analysis of ACP specific activity in the cultures of Δput4.2-GS115 and control tr2-1-GS115 strains after growth and methanol induction in media with ammonium sulphate or proline. The mean with SEM from four separate experiments with two technical replications is presented (* *p* < 0.05; Mann–Whitney U test).

Next, we investigated the effect of deletions in the sequences of the *KpPUT4.1*, *KpPUT4.2*, *KpGAP1.1*, *KpGAP1.2*, *KpGAP1.3* genes on the repression of the *AOX1* gene promoter by proline. *K. phaffii* strains (Δgap1.1-GS115, Δgap1.2-GS115, Δgap1.3-GS115, Δput4.1-GS115, Δput4.2-GS115) with deletions and the control strain (tr2-1-GS115) were grown on plates with minimal media containing methanol. Ammonium sulfate or proline was used as the nitrogen source. Synthesis of ACP is the result of the activity of the *AOX1* gene promoter in the reporter system. After 48 h of growth, ACP reporter activity on the surface of the colonies was determined qualitatively (Figure 5a).

For Δgap1.1-GS115, Δgap1.2-GS115, Δgap1.3-GS115 and Δput4.1-GS115 strains no significant differences were shown in the activity of the reporter gene compared to the tr2-1-GS115 control strain. ACP activity was higher on media with ammonium sulphate in comparison to medium with proline. In the Δput4.2-GS115 strain, which carries a deletion in the *KpPUT4.2* gene, the activity of the ACP reporter when cells were grown on the medium with proline is higher than the control strain tr2-1-GS115.

For qualitative analysis *K. phaffii* Δput4.2-GS115 and *K. phaffi* tr2-1-GS115 control strains were grown in 100 mL glycerol-containing media with ammonium sulfate (BMGN) or proline (BMGP). After 40 h of cultivation, the cells were collected and placed in 100 mL methanol-containing media with ammonium sulfate (BMMN) or proline (BMMP). After 40 h of methanol induction, the reporter ACP activity was measured (Figure 5b). Hence, we report that *KpPUT4.2* gene deletion in *K. phaffii* leads to the partial removal of the repressive proline effects on the *AOX1* promoter.

## 4. Discussion

Promoters of *MUT* genes, especially *AOX1* promoter, are often used for heterologous gene expression in *K. phaffii*. Hence, their regulation and its molecular mechanisms are actively being studied. Previously it was shown that these promoters are repressed when the culture medium contains certain amino acids, especially proline [17,18].

To analyze the effects of proline on gene expression in *K. phaffii*, RNA-sequencing was performed. The experiment setup was based on cultivation strategy, which is often used for the production of recombinant proteins. In the first stage, biomass of cells was grown in media with glycerol and ammonium sulphate or proline. The second stage was the induction in media with methanol and ammonium sulphate or proline.

Transcriptome analysis revealed drastic changes in gene expression when proline was present in the media in comparison with media containing ammonium sulphate. Among 4920 protein encoding genes, 929 (18.9%) were differentially expressed in experimental conditions; 554 genes (~11%) demonstrated increased expression in media with proline; and 375 genes (~8%) genes demonstrated a decrease in expression levels.

It was shown that expression of known *MUT* genes, which are involved in all stages of methanol metabolism, is suppressed at the transcriptional level when proline is present in the media. mRNA levels decreased both for the genes involved in the dissimilative and the assimilative branch of methanol metabolic pathway. Such repression of *MUT* genes may be connected with the fact that *K. phaffii* is capable of using proline [20] and some other amino acids [19] as the only carbon, energy and nitrogen source.

In this study, we analyzed the activity of the *KpPUT1* gene, which presumably encodes proline oxidase—the first enzyme involved in proline utilization. It was shown, that the promoter of this gene is active when *K. phaffii* is grown on media with proline and different carbon sources. Moreover *KpPUT1* gene promoter is active even when *K. phaffii* is grown on the media with proline and ammonium sulphate as nitrogen sources. Thus, *K. phaffii* cells catabolize proline even in the presence of other carbon and energy sources (glycerol, glucose or methanol) and ammonium sulphate.

Based on the data of transcriptome analysis it may be proposed that in *K. phaffii* cells proline is actively oxidized in mitochondria. The resulting glutamate is converted to α-ketoglutarate by glutamate dehydrogenase. Interestingly, a GABA shunt is also very active providing synthesis of succinate. Both α-ketoglutarate and succinate enter the TCA cycle. The balance of the reactions seems to be shifted to production of oxaloacetate. It is transformed into phosphoenolpyruvate by phosphoenolpyruvate carboxykinase, which is the first key enzyme in gluconeogenesis.

Active use of proline as a carbon source by *K. phaffii* explains the suppression of genes involved in assimilative branch of methanol metabolic pathway. On the other hand proline catabolism also provides *K. phaffii* cells with energy. Previously it was found that proline in the media provided stronger repression of the *AOX1* gene promoter than glutamate [17]. Oxidation of proline to glutamate provides energy for the cell, which explains the suppression of *MUT* genes involved in dissimilative branch of the methanol metabolic pathway. Thus, molecular mechanisms of the repression of *MUT* genes should be intertwined with the regulation of carbon and energy metabolism in *K. phaffii*.

Here, we found that expression of *MIG2* gene, which is involved in the repression of *AOX1* and other *MUT* genes [10] is increased when *K. phaffii* is cultured in proline-containing media. In *S. cerevisiae* yeast, the transcriptional repressor Mig1 controls the expression of genes involved in metabolism and the transport of alternative carbon sources. This protein participates in Snf1 kinase signaling pathway. In this pathway Snf1 kinase activity is regulated by any of three upstream kinases Sak1, Tos3 and Elm1 [51]. Previously it was shown that KpSnf1 and XP_002491563.1 kinase (encoded by *PAS_chr2-1_0639*) that is orthologous to Sak1, Tos3 and Elm1 in *S. cerevisiae* are important kinases mediating *AOX1* promoter activation by methanol [14]. Here, we demonstrate that *PAS_chr2-1_0639* is activated in medium containing proline. Therefore, we propose that *K. phaffii MIG2* and *PAS_chr2-1_0639* genes are involved in the regulation of *MUT* genes by proline. Their role will be investigated in further studies.

In this study we analyzed the role of the genes encoding membrane transporter proteins *KpGAP1.1*, *KpGAP1.2*, *KpGAP1.3*, *KpPUT4.1*, and *KpPUT4.2* in the regulation of the *AOX1* gene promoter. It was found that deletion in the *KpPUT4.2* gene leads to the partial derepression of the *AOX1* gene promoter in media with methanol and proline. Results of the transcriptome analysis demonstrate that *KpPUT4.2* expression is highly induced in media with proline.

*KpPUT4.2* participation in the regulation of the *AOX1* gene may be indirect. It can be assumed that the key factor triggering the repression of the *AOX1* gene is the intracellular concentration of proline and/or its active utilization. In this case, removal of the specific proline transporter encoded by the *PpPUT4.2* gene results in lower concentrations of proline within the cell. This, in turn, results in *AOX1* gene derepression. However, at this stage it is not possible to rule out the possibility that the *PpPUT4.2* gene encodes the proline sensor or a transceptor protein.

The results obtained in this study are of practical importance due to the fact that most of the biotechnological media used for *K. phaffii* cultivation include peptone and yeast extract which contain proline and other amino acids. The addition of proline to culture medium may be used as a way to fine-tune recombinant gene expression, especially when the synthetized protein is toxic for *K. phaffii* cells.

On the other hand, in recent years *K. phaffii* has become a new model organism for fundamental research [52]. Its extensive study and comparison with such established models as *S. cerevisiae* will provide us with insights on the process of evolution of metabolic and regulatory systems in nature.

Nitrogen catabolite repression (NCR) in *S. cerevisiae* cells is activated when good nitrogen sources, such as glutamine or ammonium salts, are present in the medium. It results in downregulation of genes involved in utilization of poorer nitrogen sources, such as proline [53]. In the first studies on NCR, mainly glucose was used as a carbon source. For Crabtree-positive yeast *S. cerevisiae* high levels of glucose may inhibit respiration and activate fermentation processes. On the other hand, proline utilization requires the activity of mitochondrial enzymes. Therefore inhibition of mitochondria during fermentation may be the reason why proline metabolism is a target of NCR in *S. cerevisiae*.

The analysis of the activity of the *KpPUT1* proline oxidase gene promoter demonstrates that proline is catabolized by *K. phaffii* cells even in the presence of a good nitrogen source—ammonium sulphate. Thus, regulation of nitrogen metabolism in *K. phaffii* and *S. cerevisiae* is different. This can be explained by the fact that *K. phaffii* yeast is Crabtree-negative and depends on respiration. In this study using an MTT assay, we demonstrated that mitochondrial activity in *K. phaffii* cells increased during growth in the medium containing methanol and proline in comparison to the medium containing methanol and ammonium sulphate.

## Figures and Tables

**Table 1:** *K. phaffii* strains used in this study.

Strain	Genotype	Sourse
GS115	*his4*	Thermo Fisher Scientific, Waltham, MA, USA
X-33	*wt*	Thermo Fisher Scientific, Waltham, MA, USA
tr2-1-GS115	*phox PAOX1-PHO5 HIS4*	17, 18
P1AP-GS115	*PPUT1-PHO5 HIS4*	This study
Δgap1.1-GS115	Δ*kpgap1.1 ZeoR PAOX1-PHO5 HIS4*	This study
Δgap1.2-GS115	Δ*kpgap1.2 ZeoR PAOX1-PHO5 HIS4*	This study
Δgap1.3-GS115	Δ*kpgap1.3 ZeoR PAOX1-PHO5 HIS4*	This study
Δput4.1-GS115	Δ*kpput4.1 ZeoR PAOX1-PHO5 HIS4*	This study
Δput4.2-GS115	Δ*kpput4.2 ZeoR PAOX1-PHO5 HIS4*	This study

## Data Availability

Not applicable.

## Supplementary Materials

The following supporting information can be downloaded at: https://www.mdpi.com/article/10.3390/microorganisms10010067/s1, Figure S1: PCR analysis of *K. phaffii* strains, obtained in the study; Figure S2: The analysis of the phenotypes of *K. phaffii* strains carrying the deletions in permease genes; Table S1: Sequences of primers used in the study; Table S2: Number of reads. Table S3: The results of differential gene expression analysis using DESeq2; Table S4: Differentially expressed genes discussed in the paper; Table S5: Results of protein sequences alignments.
