# Supplementary material for: Transcriptome Analysis Unveils the Effects of Proline on Gene Expression in the Yeast Komagataella phaffii
Source: Microorganisms. 2021 Dec 29;10(1):67. doi: 10.3390/microorganisms10010067 (PMC8778476; doi:10.3390/microorganisms10010067)
Supplement: Supplementary file 1 [file microorganisms-10-00067-s001.zip › Supplementary/Figure S1. PCR analysis of K. phaffii strains, obtained in the study.pdf]

Figure S1. PCR analysis of *K. phaffii* strains, obtained in the study

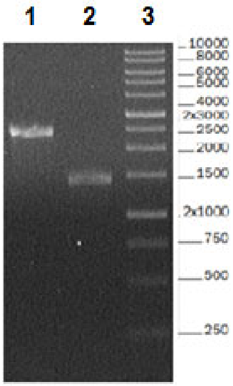

Figure S1a. Electropherogram of the results of PCR with genomic DNA of the *K. phaffii* P1AP-GS115 strain: lane 1 – PCR with primers PPUT1-F-SacI and PHO5-R-AvrII, fragment size ~2360 bp, lane 2 - PCR with primers PHO5-F and PHO5-R-AvrII, fragment size ~1420 bp, lane 3 – 1kb DNA ladder (Evrogen, Russia).

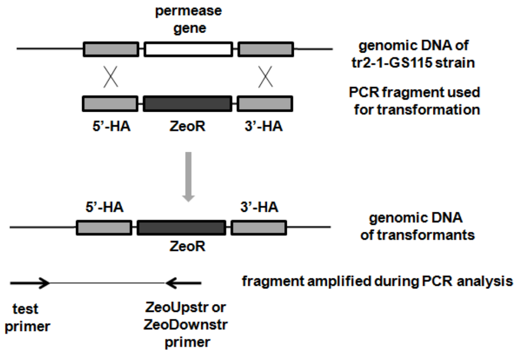

Figure S1b. Scheme representing the transformation of the tr2-1-GS115 strain during introduction of deletions in permease genes. Position of test primers used for PCR analysis of *K. phaffii* Δgap1.1-GS115, Δgap1.2-GS115, Δgap1.3-GS115, Δput4.1-GS115 and Δput4.2-GS115 strains is shown. Use of ZeoUpstr or ZeoDownstr depended on the orientation of zeocin resistance (ZeoR) gene in PCR fragment used for transformation.

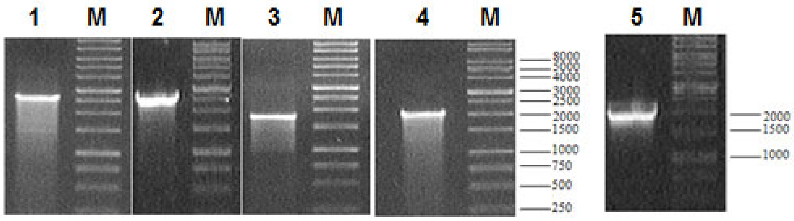

Figure S1c. Electropherogram of the results of PCR with genomic DNA of the *K. phaffii* strains: lane 1 – PCR with primers testGAP1.1 and ZeoDownstr and genomic DNA of the Δgap1.1-GS115 as the template, fragment size ~2500 bp; lane 2 – PCR with primers testGAP1.2 and ZeoDownstr and genomic DNA of the Δgap1.2-GS115 as the template, fragment size ~2430 bp; lane 3 – PCR with primers testGAP1.3 and ZeoUpstr and genomic DNA of Δgap1.1-GS115 as the template, fragment size ~1700 bp; lane 4 – PCR with primers testPUT4.2 and ZeoUpstr and genomic DNA of the Δput4.2-GS115 strain as the template, fragment size ~1700 bp; lane 5 – PCR with primers testPUT4.1 and ZeoUpstr and genomic DNA of the Δput4.1-GS115 strain as the template, fragment size ~1800 bp; lanes M – 1kb DNA ladder (Evrogen, Russia).
