# Supplementary material for: Transcriptome Analysis Unveils the Effects of Proline on Gene Expression in the Yeast Komagataella phaffii
Source: Microorganisms. 2021 Dec 29;10(1):67. doi: 10.3390/microorganisms10010067 (PMC8778476; doi:10.3390/microorganisms10010067)
Supplement: Supplementary file 1 [file microorganisms-10-00067-s001.zip › Supplementary/Figure S2. The analysis of the phenotypes of K. phaffii strains carrying the deletions in permease genes.pdf]

The analysis of the phenotypes of *K. phaffii* strains carrying the deletions in permease genes

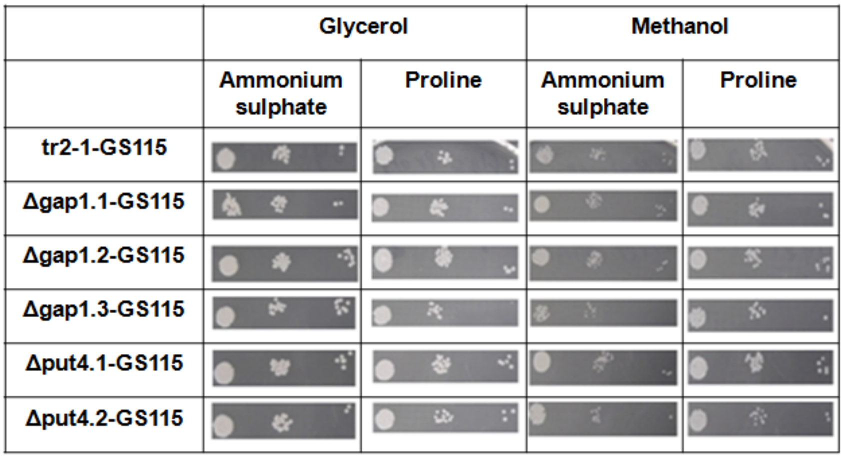

Figure S2a. Growth of *K. phaffii* strains Δgap1.1-GS115, Δgap1.2-GS115, Δgap1.3-GS115, Δput4.1-GS115, Δput4.2-GS115 and control tr2-1-GS115 strain on media with glycerol or methanol as the main carbon source. Ammonium sulfate or proline was used as the only nitrogen source.

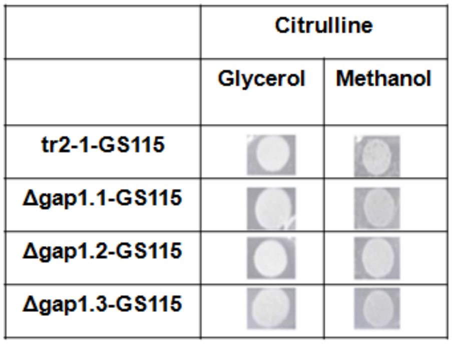

Figure S2b. Growth of *K. phaffii* strains Δgap1.1-GS115, Δgap1.2-GS115, Δgap1.3-GS115 and control tr2-4-GS115 strain on media with glycerol or methanol as carbon source. Citrulline was used as the only nitrogen source.
