# Supplementary material for: Transcriptome Analysis Unveils the Effects of Proline on Gene Expression in the Yeast Komagataella phaffii
Source: Microorganisms. 2021 Dec 29;10(1):67. doi: 10.3390/microorganisms10010067 (PMC8778476; doi:10.3390/microorganisms10010067)
Supplement: Supplementary file 1 [file microorganisms-10-00067-s001.zip › Supplementary/Table S1. Sequences of primers used in the study.pdf]

Table S1. Sequences of primers (5′-3′) used in the study

Name	Sequence
ZeoDownstr	AGTTGACCAGTGCCGTTC
ZeoUpstr	CGGCGTACAGCTCGTC
KpGAP1.1-5F	CTCGAGATTCACAAGGCTGCATTAGA
KpGAP1.1-5R	CCCAGGATCCAAATCAACTTAGCGGTCCAT
KpGAP1.1-3F	ATTTGGATCCTGGGTACTGAAGTGTGTGG
KpGAP1.1-3R	CTCGAGGTTTTCTAATTGCTCGTGCT
KpGAP1.2-5F	CCCTAGGAGGAATCCGATCTAGAGA
KpGAP1.2-5R	GCACGGATCCGTTTGCGACTGGTGATTTAC
KpGAP1.2-3F	AAACGGATCCGTGCTGATCTTTGTAGTGACC
KpGAP1.2-3R	CCTAGGTGAGTAAGGAGGGTAGGAAG
KpGAP1.3-5F	CTCGAGTGAGGTCGCTCTTTAGTCTG
KpGAP1.3-5R	CCAGGGATCCTCATTATAAGATAGTTGTGTTGACG
KpGAP1.3-3F	ATGAGGATCCCTGGCTGTTAGCTCTCTCC
KpGAP1.3-3R	CTCGAGCGTGACTTTATGCTGTGTGT
KpPUT4.1-5F	CTCGAGCGACTATCGGTCTTGTTAGG
KpPUT4.1-5R	ATAAGGATCCCTGTGGAGAGGAAGTCAAAC
KpPUT4.1.3F	CAGGGATCCTTATTGCTGCTTATGTTACTATCC
KpPUT4.1.3R	CTCGAGAATGTGCCCAAGACGAC
KpPUT4.2-5F	CTCGAGCCCATACACTTGTCGTAAACT
KpPUT4.2-5R	GAATGGATCCCAGTCAGATCATTAAAAGCCA
KpPUT4.2-3F	CTGGGATCCATTCCTAAGGTCGCTTCATC
KpPUT4.2-3R	CTCGAGCTACGGAGGGAAAGAAAGAC
testKpGAP1.1-5	AGTTAGCCCAATGTCAAGAA
testKpGAP1.2-5	AAACGACCTCAGAGACAGAA
testKpGAP1.3-5	TACGGATAACGACACTGTGA
testKpPUT4.1-5	GTGTCAGATATCACGTGCAG
testKpPUT4.2-3	AGATCAACTGGATCGTCAAC
PPUT1-F-SacI	ATTACAGAGCTCTACGCATCTGCTATGGA
PPUT1-R	GAATAAACAACAGATTTAAACATTTATGACTGTATTACTG AGTAATAAA
PHO5-F	ATGTTTAAATCTGTTGTTTATTCAA
PHO5-R-AvrII	ATTACACCTAGGAAAACTATTGTCTCAATAGACTGG
