# Supplementary material for: Transcriptome Analysis Unveils the Effects of Proline on Gene Expression in the Yeast Komagataella phaffii
Source: Microorganisms. 2021 Dec 29;10(1):67. doi: 10.3390/microorganisms10010067 (PMC8778476; doi:10.3390/microorganisms10010067)
Supplement: Supplementary file 1 [file microorganisms-10-00067-s001.zip › Supplementary/Table S2. Number of reads.pdf]

Table S2. Number of reads for each sample analyzed by RNA-sequencing

Sample	Medium (biomass growth / induction)	Number of reads
N1	BMGN / BMMN	449 310
N2	BMGN / BMMN	569 501
N3	BMGN / BMMN	831 178
P1	BMGP / BMMP	432 625
P2	BMGP / BMMP	487 735
P3	BMGP / BMMP	648 008
